# The Role of Public Health in Promoting Quality Improvement in Care for Stroke and Heart Disease

**Published:** 2008-03-15

**Authors:** Mary G George, Michael D Matters, Jipan Xie, Henraya F McGruder, Amy L Valderrama

**Affiliations:** Division for Heart Disease and Stroke Prevention, National Center for Chronic Disease Prevention and Health Promotion, Centers for Disease Control and Prevention; Northrop Grumman Information Technology, Atlanta, Georgia; Northrop Grumman Information Technology, Atlanta, Georgia; Division for Heart Disease and Stroke Prevention, National Center for Chronic Disease Prevention and Health Promotion, Centers for Disease Control and Prevention, Atlanta, Georgia; Division for Heart Disease and Stroke Prevention, National Center for Chronic Disease Prevention and Health Promotion, Centers for Disease Control and Prevention, Atlanta, Georgia

The goals of stroke care are to reduce the incidence of, and illness and death from, stroke while improving quality of life for stroke survivors. Stroke systems of care coordinate and promote access to optimal care from the identification, reduction, and treatment of risk factors through prevention of recurrent stroke and rehabilitation; promote changes in hospital policies and systems to improve delivery of care; and ultimately improve patient outcomes. Traditionally public health has not focused on quality of care (QoC) for acute events of a chronic disease, such as stroke. Yet, through the core functions of public health — assessment, policy development, and assurance (1) — state health departments are uniquely positioned to foster quality improvement (QI) for care of stroke and heart disease patients. State health departments' expertise in surveillance can be used for QoC and QI activities, enabling them to translate research into programs that assess and improve QoC for stroke patients, resulting in development of necessary components of stroke systems of care, and ultimately ensuring patient access to high-quality stroke care.

## State Public Health Departments' Role in Improving Quality of Care

State public health agencies conduct a variety of activities that can improve QoC for stroke and heart disease. These include reporting state-mandated statistics for measuring QoC, such as in-hospital death rates for stroke (2) and death rates for heart surgery. Continuous QI — a cycle of collecting information on QoC, then using that information to continually improve care — requires measurement and tracking of QoC at both the individual patient level and the population level.

### Paul Coverdell National Acute Stroke Registry

In 2001, Congress provided funding to the Centers for Disease Control and Prevention (CDC) for the Paul Coverdell National Acute Stroke Registry (PCNASR) to implement state-based stroke registries. These registries track and measure QoC for stroke patients from prehospital emergency medical services (EMS) through acute care, secondary prevention, and rehabilitation, and they promote changes in hospital policies and systems to improve delivery of care (3). After prototype testing, CDC funded four state health departments for 3 years to establish the PCNASR in their states. State public health departments implement the PCNASR and foster QI by collaborating with physicians, hospital management, hospital QI teams, and others striving to improve stroke care. Similar to the PCNASR QI initiatives, efforts are under way to improve the QoC and response times of EMS for people with acute symptoms of stroke and heart disease. Integration of QI within state health departments for stroke care will increase opportunities to develop statewide systems of stroke care. Integration of stroke-related activities of EMS, hospitals, clinicians, and other providers of stroke care should improve access to care and ultimately refine policies and stroke systems of care.

State health departments continuously evaluate their QI interventions (e.g., dysphagia screening to prevent aspiration pneumonia and initiation of secondary prevention) that improve care for stroke patients. Results from the prototype registries identified gaps in acute care for stroke, underscoring the importance of continuously monitoring QoC indicators and the need for QI interventions (4).

### Trauma systems

During the past decade, federal agencies collaborated with the American College of Surgeons and other partners to oversee development of trauma care registries to improve QoC for both individual trauma patients and systems of trauma care. This effort is an essential component of effective statewide trauma care. Because trauma is an important public health problem (5,6), the National Trauma–EMS Stakeholder Group, directed by the Health Resources and Services Administration (7), recently adopted a public health approach for developing trauma systems, allowing for the inclusion of trauma care professionals, public health officials, and health care policy experts. State health departments have contributed to assessment of both patient-level QoC and the trauma system itself through the three core functions of public health.

## Public Health and Quality Improvement in Stroke Care

### Assurance through leadership

The Institute of Medicine (IOM) recommended that state health departments' public health duties (1) include "assessment of health needs within the state based on statewide data collection," "assurance of an adequate statutory base for health activities in the state," and "assurance of appropriate organized statewide effort to develop and maintain requisite personal, educational, and environmental health services." QI in stroke care is a multidisciplinary, systems-focused effort engaging public and private entities and involving a wide spectrum of care. Because state health departments have considerable influence over their states' health care budget, policies, and regulations, they are key in providing leadership in QI through policy development and assurance. The IOM-stated public health duties have practical implications for QI in health care. The IOM recommendations and QI experience in public health (8) illustrate the leadership role of state health departments in ensuring QI of stroke care.

#### Create the culture and environment to foster a statewide QI effort

One effective way to improve QoC is to share QI interventions and the outcomes of these interventions. State health departments can lead this effort through a collaborative model of communication with providers of care. In states participating in the PCNASR, state public health departments coordinate the overall effort to improve QoC for stroke through the program's state QI directors.

#### Build capacity for conducting QI activities

Continuous QI requires substantial resources and commitment from state and local health departments, EMS systems, individual providers in the chain of stroke care, and communities. State health departments can coordinate these entities, as in North Carolina through the North Carolina Collaborative Stroke Registry and the North Carolina Office of Emergency Medical Services.

#### Develop policies and plans to promote and support evidence-based QI activities

State health departments play a leading role in developing and implementing policies to improve health care. Using evidence-based practice, CDC, The Joint Commission, and the American Heart Association/American Stroke Association have developed new QI measures in stroke care. Putting new guidelines into practice in the health care system relies on the states' leadership in integrating new information into their policies and plans.

#### Enforce laws and regulations to ensure quality of statewide stroke care

State leadership is irreplaceable in advocating for evidence-based health policy. State health departments should be powerful advocates of new regulations to improve QoC and are the agencies primarily responsible for implementing these regulations. For example, the Coordinated Quality Improvement Program was created under the Washington State Department of Health during the 1993 legislative session; the New York State Health Information and Quality Improvement Act was enacted and signed into law in New York in 2000; and both Massachusetts and New York have state-level legislation regarding certification of primary stroke centers.

### Policy development through collaboration

One of the most effective ways to improve care for stroke is to engage and continually collaborate with critical stakeholders (9) (Table). State health departments can evaluate new advances in stroke care and assess pertinent issues in systems of stroke care. Such prevention efforts should include primordial prevention (reduction of risk factors), primary prevention (identification and treatment of risk factors), and secondary prevention (prevention of recurrent stroke) (10).

The ultimate goal of engaging stakeholders in stroke care is to implement effective policies, practices, and programs that prevent stroke, control risk factors, and improve the QoC and outcomes of stroke patients. State health departments should further this goal by implementing stroke programs that increase awareness of symptoms and risk factors for stroke while measuring disease burden within the state.

### Assessment and evaluation — a cornerstone of stroke systems of care

The American Stroke Association's Task Force on the Development of Stroke Systems has developed recommendations for establishing systems of stroke care (10), and public health agencies at all levels support many of these recommendations. Three examples of these key components of stroke systems of care and the ways in which public health helps to develop them follow.

#### Notification and response of EMS

The North Carolina EMS Stroke Toolkit Project (11), sponsored by CDC, is a collaboration between the National Association of Chronic Disease Directors, the state EMS program, and the University of North Carolina, Chapel Hill. This Web-based QI program works with North Carolina's electronic records management system — mandated for all 100 county-based EMS departments — to generate reports, tables, and graphs for all stages of EMS response to stroke (i.e., dispatch, travel and transport mode and times, diagnosis, EMS treatment, and an on-site interview). EMS can use the toolkit to reduce time to transport patients with possible stroke.

#### Treatment of stroke

The PCNASR (12) collects data on care provided to patients with stroke, identifies appropriate evidence-based performance measures for QI, and uses continuous QI strategies at the hospital level. By tracking care using evidence-based measures of optimal stroke care, such as screening for dysphagia or assessment for rehabilitation, it fulfills a critical function of stroke systems of care: tracking, measuring, and improving quality of care (10).

#### Subacute treatment of stroke and secondary prevention

The PCNASR uses evidence-based standards, such as screening for dysphagia, prophylaxis for deep vein thrombosis, and initiation of secondary prevention, to prevent common complications of stroke, thus fulfilling the functions of assessment and assurance.

These projects require use of public health informatics to improve the flow of information among different parts of the health information system. We anticipate a growing need for state health departments to monitor QoC by using agile data systems based on a solid public health informatics infrastructure. State health departments already engaged in developing key components of stroke systems of care are strategically positioned to integrate these components.

### Future Directions

PCNASR provides a model for other public health efforts to improve the QoC for cardiovascular diseases, such as acute coronary syndrome and heart failure. In 2004, the American Heart Association updated its guidelines for managing ST-elevation myocardial infarction (STEMI) (13) and, more recently, updated recommendations for developing systems of care for acute coronary syndrome, which include a role for public health (14).

In 2004, the Organized Program to Initiate Lifesaving Treatment in Hospitalized Patients with Heart Failure (OPTIMIZE-HF) registry examined use of performance measures for improving QoC for patients with heart failure. Adherence to performance measures did not correlate well with death or rehospitalization performance measures for heart failure in 259 U.S. hospitals (15,16), indicating a need for better methods or different performance measures to assess and improve care of patients with heart failure (16). Nevertheless, the increasing numbers of patients with heart disease, the chronic nature of their disease, the associated high costs, and frequent hospitalizations reinforce the need to evaluate and promote QoC in this area.

The PCNASR aims to integrate the stroke-related activities of EMS, hospitals, and providers of stroke care to promote stroke systems of care and to enhance QoC for stroke patients. Integration of these services and providers could extend to QoC for acute coronary syndrome and heart failure patients in developing QI-focused systems of care for these diseases as well. State public health departments can coordinate efforts to improve QoC for these cardiovascular diseases.

## Figures and Tables

**Table.** Stakeholders in Acute Stroke and Their Focus Areas

**Stakeholders**	**Task or Focus Area**
Scientists	Present and review articles in journals, and review data that detail vital findings related to stroke systems of care.
Health care providers (i.e., persons with direct patient contact)	Implement evidence-based practices of best care.
Emergency responders	Prioritize calls of patients with signs and symptoms of stroke.
Stroke organizations (e.g., American Heart Association/American Stroke Association, National Stroke Association, Brain Attack Coalition, Stroke Belt Consortium)	Improve care through research, education, advocacy, and development of science-based standards.
Local, state, and federal governments (state health departments, Centers for Disease Control and Prevention [CDC], National Institutes of Health)	Develop initiatives in stroke prevention and quality of care.
Agencies that produce national health data (e.g., CDC's National Center for Health Statistics, National Institutes of Health's Division of Populations and Prevention Services)	Produce up-to-date data on prevalence, incidence, burden, and mortality.
National advocacy organizations (e.g., Brain Attack Coalition, National Stroke Association, American Heart Association/American Stroke Association)	Initiate legislative activities, and educate legislators on the public health impact and challenges of stroke.
Professional and nonprofit organizations (The Joint Commission, American Stroke Association)	Develop initiatives in quality of care.
Community initiatives and organizations (CDC's Racial and Ethnic Approaches to Community Health)	Eliminate racial and ethnic disparities in health (e.g., cardiovascular disease).
Economists	Evaluate dollars saved by improving quality of stroke care (e.g., by reducing costs for care associated with recurrent events).
Pharmaceutical companies	Provide products for patients hospitalized with stroke.
Media outlets	Provide public service announcements that emphasize stroke education for all age groups, including children.
Organizations in areas related to stroke (e.g., physical activity, nutrition, tobacco use, alcohol use, hypertension, hypercholesterolemia, atrial fibrillation)	Partner with organizations that address multiple risk factors to broaden effective prevention efforts in public health.

## References

[1] (1988). Institute of Medicine. The future of public health.

[2] (2005). 2005 Oregon hospital quality indicators.

[3] Labarthe DR, Biggers A, LaPier T, George MG (2006). The Paul Coverdell National Acute Stroke Registry (PCNASR): a public health initiative. Am J Prev Med.

[4] Reeves MJ, Broderick JP, Frankel M, LaBresh KA, Schwamm L, Moomaw CJ (2006). The Paul Coverdell National Acute Stroke Registry: initial results from four prototypes. Am J Prev Med.

[5] Cooper G, Laskowski-Jones L (2006). Development of trauma care systems. Prehosp Emerg Care.

[6] Sanddal N Model trauma systems planning and evaluation. HSB/EMS Webcast transcript; March 27, 2006.

[7] (2006). Model trauma system planning and evaluation.

[8] Johnson KA, Little GA (1999). State health agencies and quality improvement in perinatal care. Pediatrics.

[9] (2001). Updated guidelines for evaluating public health surveillance systems: recommendations for the guidelines working group. MMWR Recomm Rep.

[10] Schwamm LH, Pancioli A, Acker JE, Goldstein LB, Zorowitz RD, Shephard TJ (2005). Recommendations for the establishment of stroke systems of care: recommendations from the American Stroke Association's Task Force on the Development of Stroke Systems. Stroke.

[11] PreMIS.

[12] The Paul Coverdell national Acute Stroke Registry.

[13] Sivarajan Froelicher, Miller NH, Christopherson DJ, Martin K, Parker KM, Amonetti M (2004). High rates of sustained smoking cessation in women hospitalized with cardiovascular disease: the Women's Initiative for Nonsmoking (WINS). Circulation.

[14] Jacobs AK, Antman EM, Faxon DP, Gregory T, Solis P (2007). Development of systems of care for ST-elevation myocardial infarction patients: executive summary. Circulation.

[15] Gheorghiade M, Abraham WT, Albert NM, Greenberg BH, O'Connor CM, She L (2006). Systolic blood pressure at admission, clinical characteristics, and outcomes in patients hospitalized with acute heart failure. JAMA.

[16] Fonarow GC, Abraham WT, Albert NM, Stough WG, Gheorghiade M, Greenberg BH (2007). Association between performance measures and clinical outcomes for patients hospitalized with heart failure. JAMA.

